# No “doom” in chicken domestication?

**DOI:** 10.1371/journal.pgen.1008089

**Published:** 2019-05-30

**Authors:** Mirte Bosse

**Affiliations:** 1 Wageningen University & Research, Animal Breeding and Genomics, Wageningen, the Netherlands; 2 Department of Ecological Science, Animal Ecology Group, Vrije Universiteit Amsterdam, Amsterdam, the Netherlands; University of Bern, SWITZERLAND

Domestication has generally been associated with certain genetic costs for the species that has been domesticated. This “cost of domestication” was first described in plants [1], and this view has also been adopted for domestic animals, such as horses [2] and dogs [3]. The costs that are referred to include an elevated number of harmful mutations in the genomes of the domestic lineages compared with their wild ancestors. The reasons for this cost are a reduced efficiency of selection against mildly deleterious alleles due to bottlenecks, linked selection with preferred alleles, and allelic surfing during expansion [4, 5].

Deleterious alleles are generally present at a low frequency [6]. Small populations are more prone to drift, and less efficient selection is predicted to allow mildly deleterious mutations to drift to a higher frequency. According to theory, therefore, small domestic populations should have lower fitness because their genomes carry an increased mutational load, and the harmful variants are more often in a homozygous state [7]. Prediction algorithms of deleteriousness using sequence data indeed corroborate the classical theory about the population frequency of deleterious alleles (e.g., [8]). Numerous examples have emerged using such predictions from sequence data, in which genomes from endangered species in the wild indeed displayed reduced genetic variation and had accumulated deleterious mutations (gorilla [9], island fox [10]). Inbreeding and the loss of potentially beneficial alleles due to drift can cause problems in the long term for populations that experienced a bottleneck at some point in their demographic history. Moreover, harmful mutations have a chance to rise to higher frequencies through drift. A substantial debate exists about whether ancient and recent bottlenecks had a strong influence on the occurrence of deleterious alleles in human populations [11, 12, 13]. However, in line with the costs of domestication hypothesis, in domesticated animals, it is generally assumed that the bottleneck during domestication resulted in higher mutational load [14, 15]. Nevertheless, several recent studies indicate that domestication is not necessarily accompanied by a strong bottleneck but, instead, has often occurred over long periods, with extensive gene flow between wild and domestic populations [16, 17]. The more recent establishment of defined breeds in many domestic animals, with dog breeds as an extreme example, most likely provided the main bottlenecks resulting in the observed lower genetic diversity and higher mutational load in domestic populations. In chickens, this is most clearly seen in the layer lines, in particular, the white leghorn type of breeds.

**Fig 1:**
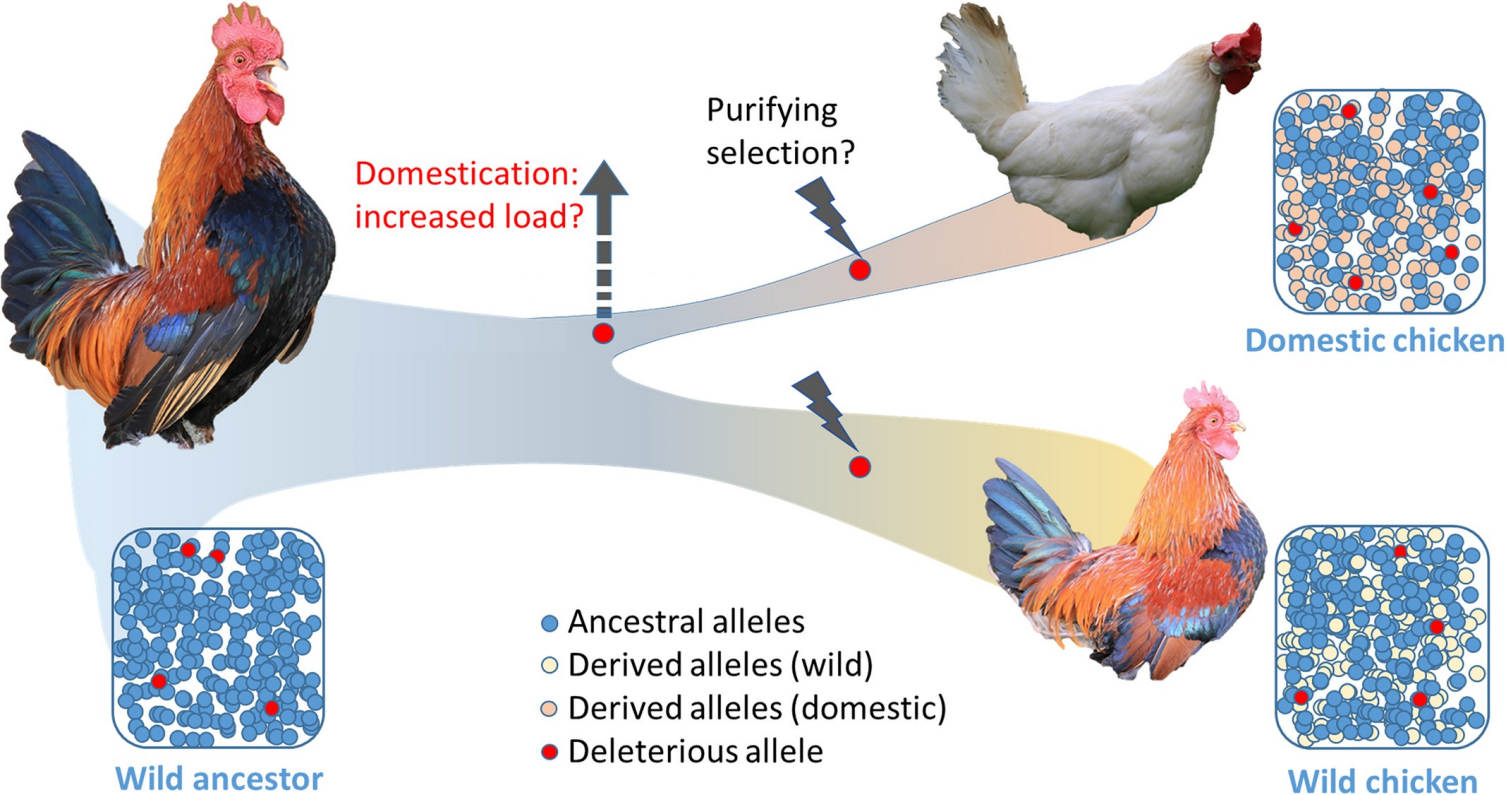
Schematic overview of chicken domestication and selection. As reported by the study in [22], the alleles that differ strongly in allele frequency between domestic and wild chickens show a deficit in potentially deleterious alleles. This indicates that deleterious alleles were successfully selected against during the trajectory of domestication and breed formation and did not rise in frequency in their wild counterparts either. However, which process contributed most to current differences between wild and domestic chickens and when purging occurred remain open questions.

Chicken were domesticated from red jungle fowl some thousands of years ago during the Neolithic period [18, 19]. Genome sequencing has enabled the study of the genomic consequences of chicken domestication [20, 21]. In a recent study in *PLOS Genetics* [22], the authors investigated allele frequency shifts during domestication and selection in the commercial chicken, using over 20 million SNPs derived from whole-genome sequences from 127 animals. The authors reported that the differentiation between wild and domestic chickens is mild at most loci, with only a few strongly differentiated regions, potentially due to hard selective sweeps. These results indicate that, even though breed- or population-specific drift can be high, the overall differentiation between wild and domestic chickens seems to be modest. Moreover, the loci that are highly differentiated between wild and domestic chickens significantly lack missense mutations, which is indicative of purifying selection. Interestingly, the alleles with potential functional consequences often lie in genes of commercial interest (i.e., affecting a commercially important trait). This implies that these alleles have risen to high frequency by intent, because of artificial selection, as suggested by Derks and colleagues [23]. Such missense mutations with a potential beneficial effect on egg laying can be found in the chicken thyroid stimulating hormone receptor (*TSHR*, [21]). Fig 1 summarizes the potential genomic consequences of domestication and artificial selection in chickens (Fig 1).

Although drift and inbreeding can be problematic for small populations, according to this study, they seem to be less of a problem in the domestic chicken pool, since selective sweeps or regions of high differentiation are narrow, spread out over the genome, and involved in different characteristics. Furthermore, only a few regions displayed high differences in allele frequencies, indicating that a large part of the ancestral variation is still present in domestic chickens. However, commercial lines contain many fewer rare alleles, which is indicative of a recent bottleneck, as was also found by Muir and colleagues [24]. Some important questions arise after reading the conclusions of that paper:

First, what did the variation landscape look like in the ancestral lineages prior to and after domestication? The authors use contemporary red jungle fowl genomes as a proxy for the ancestors. Are the current substitutes for wild ancestors representative? One red jungle fowl population used is highly inbred, and both red jungle fowl populations have exhibited some gene flow from commercial chickens. We have seen in gorillas and horses that ancient genomes might have fewer deleterious alleles [25, 2]. In addition, some alleles in domestic chickens might have risen in frequency only recently, such as the *TSHR* allele [26].

Second, during which stage of the domestication and selection continuum of the commercial chicken was purging most apparent? In some wild populations, purging might have counteracted genome degradation by removing harmful mutations, as has been suggested for the island fox [9]. What implication does this have for the potential to remove harmful mutations from current breeding lines as well as small populations under threat in the wild? Recurrent hybridization could alleviate the load in bottlenecked domestic species, as suggested by Wang and colleagues [4].

Third, the effect size of harmful mutations derived from sequence data is still hard to predict. More information on the deleteriousness of the variants under study will shed more light on the effects of purging and natural and artificial selection. Theory predicts that strongly deleterious recessive variants will become less abundant, whereas slightly deleterious variants will rise in frequency, as shown by the shape of the frequency spectra provided by Moyers and colleagues [15]. Furthermore, many mutations with a putative deleterious effect seem to be desired in the domestic setting.

Overall, the paper provides a more optimistic view on the effects of domestication and artificial selection on the genome of domesticated animals, challenging the question of how expensive the genetic costs of domestication really are.
